# Limited therapeutic efficacy of *N*-acetyl-L-leucine in a mouse model of CLN1 disease

**DOI:** 10.1038/s41598-025-32984-x

**Published:** 2025-12-29

**Authors:** Ewa A. Ziółkowska, Nicole A. Pagán Torres, Hsintsung Chen, Letitia L. Williams, Elizabeth M. Eultgen, Agnieszka Nowacka, Joshua T. Dearborn, Hemanth R. Nelvagal, Ineka T. Whiteman, Frances M. Platt, Jonathan D. Cooper

**Affiliations:** 1 Department of Pediatrics, School of Medicine, Washington University in St. Louis, 660 S Euclid Ave, St. Louis, MO 63110 USA; 2 Department of Pharmacology, University of Oxford, Mansfield Road, Oxford, OX1 3QT UK; 3 Department of NeurosurgeryCollegium Medicum in Bydgoszcz, Nicolas Copernicus University in Toruń, ul. Curie Skłodowskiej 9, 85–094 Bydgoszcz, Poland; 4 University College London, UCL Queen Square Brain Bank, G02 1 Wakefield Street, London, WC1N 1PJ UK; 5 Batten Disease Support, Research and Advocacy Foundation (US), P.O. Box 30049, Gahanna, OH 43230 USA; 6 Batten Disease Support and Research Association (Australia), 74 McLachlan Avenue, Shelly Beach, NSW 2261 Australia; 7 Beyond Batten Disease Foundation, 3305 Steck Ave., Suite 200, Austin, TX 78757 USA; 8 Department of Neurology, School of Medicine, Washington University in St. Louis, St. Louis, MO 63110 USA; 9 Department of Genetics, School of Medicine, Washington University in St. Louis, St. Louis, MO 63110 USA

**Keywords:** CLN1 disease, *N*-acetyl-L-leucine, Lysosomal storage disorder, Neurodegeneration, Motor function, Therapeutic intervention, Diseases, Neurology, Neuroscience

## Abstract

CLN1 disease, one of the most severe forms of neuronal ceroid lipofuscinosis (NCLs or Batten disease), is a rapidly progressing pediatric neurodegenerative disorder caused by mutations in the *PPT1* gene. The disease is characterized by lysosomal storage accumulation, an early onset neuroimmune response, motor impairment, and premature death. N-acetyl-L-leucine (NALL), an orally bioavailable modified amino acid, has demonstrated clinical efficacy in Niemann–Pick type C and other lysosomal storage disorders. Here, we assessed the efficacy of chronic NALL treatment in the *Ppt1*^*−/−*^ mouse model of CLN1 disease. Mice received NALL (0.1 g/kg/day) in chow either from weaning (1 month, presymptomatic) or from 4 months (symptomatic) until 7 months (normal disease endstage), with additional survival cohorts. NALL treatment did not extend survival in *Ppt1*^*−/−*^ mice and produced no significant improvement in gait coordination or rotarod performance, with only minimal improvements in select gait variability parameters in presymptomatically treated mice. Histological analyses revealed no reduction in microglial or astrocyte activation, nor in storage material accumulation, key CLN1 disease-associated phenotypes. These findings indicate that NALL monotherapy has limited therapeutic efficacy in CLN1 disease mice and suggest that its mechanisms of action may not address the underlying pathophysiology of this disorder.

**Supplementary Information:**

The online version contains supplementary material available at 10.1038/s41598-025-32984-x.

## Introduction

The neuronal ceroid lipofuscinoses (NCLs), also known as Batten disease^1–3^ are a group of prematurely fatal, autosomal recessive lysosomal storage disorders (LSDs) that primarily affect the central nervous system (CNS)^4–6^. These disorders are characterized by progressive neurodegeneration, early-onset motor and cognitive decline, speech and language impairment, sleep disturbance, vision loss, seizures, and premature death. Among the 13 NCL subtypes, CLN1 disease, historically known as infantile Batten disease, is typically the earliest onset form with the most precipitous rate of disease progression^5^. CLN1 disease is caused by mutations in the *PPT1* gene^7^, which encodes the enzyme palmitoyl-protein thioesterase-1. PPT1-deficiency impairs the removal of palmitoyl groups from lipid-modified proteins, leading to intralysosomal accumulation of autofluorescent storage material^3,7^.

In CLN1 disease, symptom onset typically occurs during infancy with rapid deterioration of previously acquired motor and cognitive function, followed by vision loss, feeding and swallowing difficulty, seizures, and early death, often in the first decade^3–6,8−9^. Despite ongoing efforts, there are currently no approved treatments for CLN1 disease, although preclinical studies have investigated enzyme replacement therapy (ERT)^10^, and multiple viral vector-mediated gene therapies^11–14^, with one AAV9-mediated therapy in early clinical investigations. However, the rapid progression of disease, the need for CNS-wide biodistribution, and challenges associated with developing these approaches have slowed the development of these strategies. This highlights an unmet need for systemically administered, safe, and broadly distributed compounds capable of modifying disease progression^3^.

*N*-Acetyl-L-leucine (NALL) is a modified amino acid that is approved by the FDA as a monotherapy for treating Niemann-Pick disease type C (NPC)^15–19^, another prematurely fatal LSD, marketed under the trade name *Aqneursa™* (levacetylleucine). This success was built in part upon initial preclinical studies in *Npc1*^*−/−*^ mice, demonstrating improved motor coordination, and reduced neuroinflammation following symptomatic and presymptomatic NALL treatment^20–22^. Subsequent clinical data from a randomized placebo-controlled trial also demonstrated that 12 weeks of NALL treatment in symptomatic NPC patients significantly improved neurological symptoms, as measured by the Scale for the Assessment and Rating of Ataxia (SARA)^23^, leading to subsequent FDA approval of *Aqneursa*^17–19^.

NALL is orally bioavailable, well-tolerated, and crosses the blood-brain barrier, with reported neuroprotective, anti-inflammatory, and metabolic effects in various neurological disease models^22–25^. Its mode of action was primarily thought to involve increasing ATP production, with consequences for both mitochondrial and lysosomal function^20,26^. More recently, though, a direct effect of NALL in stimulating transcription factor EB (TFEB), a master regulator of a network of key lysosomal expressed gene products, has been reported in vitro^15^. As such, NALL appears to be an attractive therapeutic candidate for some lysosomal diseases, and this may potentially include the NCLs.

Despite its efficacy in related lysosomal storage disorders, the therapeutic potential of NALL in or any form of NCL, has not been previously explored. For logistical reasons related to lifespan and disease severity, we chose to obtain proof of concept data for the effects of chronic oral NALL administration in NCL mice using PPT1-deficient (*Ppt1*^*−/−*^) mice, a well-characterized model of CLN1 disease^27^. These *Ppt1*^*−/−*^ mice display an early motor phenotype including progressive decline in motor coordination (rotarod and balance beam tests) and gait abnormalities, recapitulating the early clinical phenotype^3–9^. Treatment was initiated at either presymptomatic stage (1 month of age) or symptomatic stage (4 months) and continued until disease endstage at 7 months, with an additional parallel survival cohort started on treatment at each age. We assessed motor performance using rotarod and gait analysis (as used in NPC mouse studies of NALL efficacy), and neuropathological changes in the somatosensory cortex (S1BF) and thalamic relay nuclei (VPL/VPM), in addition to assessing survival. While early NALL treatment (from the presymptomatic stage) led to a modest reduction in gait variability, it did not improve coordination, glial activation, lysosomal storage burden, or survival. Moreover, treatment initiated at the symptomatic stage had no beneficial effect on any outcome measure. These findings suggest that NALL has limited therapeutic potential in CLN1 disease mice and does not influence the key pathogenic mechanisms driving disease progression.

## Materials and methods

### Animals and study design

*Ppt1*^*−/−*^ mice^27^ and wild type (WT) mice were maintained separately on a congenic C57Bl/6J background at Washington University School of Medicine. Mice were housed under controlled conditions (12 h light/dark cycle, ambient temperature 22 °C) with *ad libitum* access to food (Purina Rodent Diet 5053) and water. All procedures were approved by the Institutional Animal Care and Use Committee of Washington University in St. Louis and conducted in accordance with NIH guidelines. Animal numbers and group sizes are indicated in the corresponding figure legends.

Mice were randomly assigned to eight experimental groups stratified by genotype (WT vs. *Ppt1*^*−/−*^), treatment (placebo vs. NALL), and timing of intervention (from weaning or from 4 months of age). NALL (0.1 g/kg/day) (Catalog Number 441511, Sigma-Aldrich Inc, St. Louis, MO) was administered orally via supplemented chow starting either at postnatal day 21 (presymptomatic) or at 4 months of age (symptomatic). Placebo treated mice received the same chow without NALL supplementation. One cohort of mice (*n* = 6, per treatment group, 3 male and 3 female) were maintained on these diets until normal disease endstage at 7 months of age, when this cohort of mice was euthanized for neuropathological analysis. A second cohort of mice (*n* = 10, per treatment group, 3 male and 3 female) underwent regular assessment of behavioral performance and were maintained until they became moribund or died. These investigations adhered to ARRIVE guidelines and were conducted under protocol 24–0232, which was approved by the Institutional Animal Care and Use Committee (IACUC) at Washington University School of Medicine in St. Louis, MO.

### Survival analysis

Mice were monitored daily, and the age of death was recorded. Kaplan-Meier survival curves were generated and compared statistically using the log-rank (Mantel-Cox) test.

### Behavioral testing

#### Rotarod performance

Motor coordination and endurance were evaluated using two rotarod paradigms at 5, 6, and 7 months of age^10^. In the accelerating rotarod test, the rotation speed increased linearly from 2.5 rpm to 10.5 rpm over a 200 s period. For the continuous speed rotarod test, a constant speed of 2.5 rpm was maintained for 60 s. Each mouse underwent three consecutive trials per day across three consecutive days at each timepoint, and the average latency to fall was calculated for each animal. Both presymptomatically treated (NALL from weaning) and symptomatically treated (NALL from 4 months) *Ppt1*^*−/−*^ mice, and wild type controls, were tested at the same ages to allow for longitudinal comparison of motor performance across genotypes and treatment groups.

#### Gait analysis

Automated gait analysis was performed using the CatWalk XT system^10^. Mice were allowed to traverse the illuminated glass walkway, and gait data were automatically captured and quantified using standard CatWalk XT parameters^10^. Multiple runs were recorded for each animal to ensure consistent performance. A total of 138 physical and 107 coordination-related gait variables were extracted per mouse. Longitudinal assessment was conducted from 1 to 7 months of age.

#### Statistical analysis of gait variables

To identify disease-relevant gait parameters, a mixed-design ANOVA was initially performed on untreated wild type (WT) and *Ppt1*^*−/−*^ mice across all timepoints. Age (1–7 months) was treated as a within-subject factor, and genotype (WT vs. *Ppt1*^*−/−*^) as a between-subject factor. Interaction effects (age × genotype) and main effects were evaluated to determine which gait variables demonstrated significant age- and genotype-dependent differences. Parameters showing significant effects were selected for subsequent analysis of efficacy. To assess the efficacy of NALL treatment at disease endstage (7 months), a two-way ANOVA was performed with genotype and treatment as between-subject factors. Prior to analysis, assumptions of normality and homogeneity of variances were tested using the Shapiro–Wilk test and Levene’s test, respectively. For variables that met parametric assumptions, ANOVA was followed by Tukey’s or Bonferroni-corrected post hoc comparisons to assess treatment effects within and between genotypes (e.g., *Ppt1*^*−/−*^ placebo vs. *Ppt1*^*−/−*^ NALL). For variables that violated assumptions, non-parametric alternatives (Kruskal–Wallis test, aligned rank transform ANOVA, or Wilcoxon rank-sum tests with Holm correction) were applied. This stepwise approach enabled evaluation of whether NALL treatment elicited a statistically significant effect at 7 months of age, which gait parameters were most responsive to treatment, and whether genotype influenced the treatment response. Heatmaps displaying the statistical significance of differences in gait variables between genotypes and treatment groups were generated in R version 4.5.0 (R Core Team, 2025; https://www.r-project.org) using the tidyverse package (version 2.0.0) and patchwork package (version 1.3.2).

##### Tissue processing and immunohistochemistry

At 7 months of age, mice were deeply anesthetized and perfused transcardially with phosphate-buffered saline. Brains were removed and post-fixed, cryoprotected in 30% sucrose^28,29^. Forty µm coronal forebrain sections were cut using a Microm HM430 freezing microtome (Microm International) equipped with a Physitemp BFS-40MPA freezing stage (Physitemp, Clifton, NJ). Sections were collected into 96 well plates containing cryoprotectant solution^28–29^. A one-in-six series of coronal forebrain sections from each mouse was stained on slides using a modified immunofluorescence protocol employing TrueBlack and stained with GFAP, CD68 and SCMAS antibodies. To quantify AFSM accumulation and glial activation (GFAP + astrocytes, CD68 + microglia), thresholding image analysis was performed using slide-scanned images at 10x magnification (Zeiss AxioScan Z1)^28–29^. Contours of appropriate anatomical regions were drawn and images analyzed using Image-Pro Premier (Media Cybernetics) and thresholds selected for foreground immunoreactivity above background^28–29^.

##### Quantification and statistical analysis

All analyses were performed blinded to genotype and treatment. Statistical analyses were performed using GraphPad Prism version 10.4.1 for MacOS (GraphPad Software, San Diego, CA). A two-way ANOVA with a post-hoc Bonferroni correction was used for comparison between three groups or more. A p-value of ≤ 0.05 was considered significant. In all histograms black dots indicate the mean values of individual animals, with the number of animals used (n=) indicated beneath each histogram.

## Results

### NALL treatment does not provide any survival benefit

**Fig. 1:**
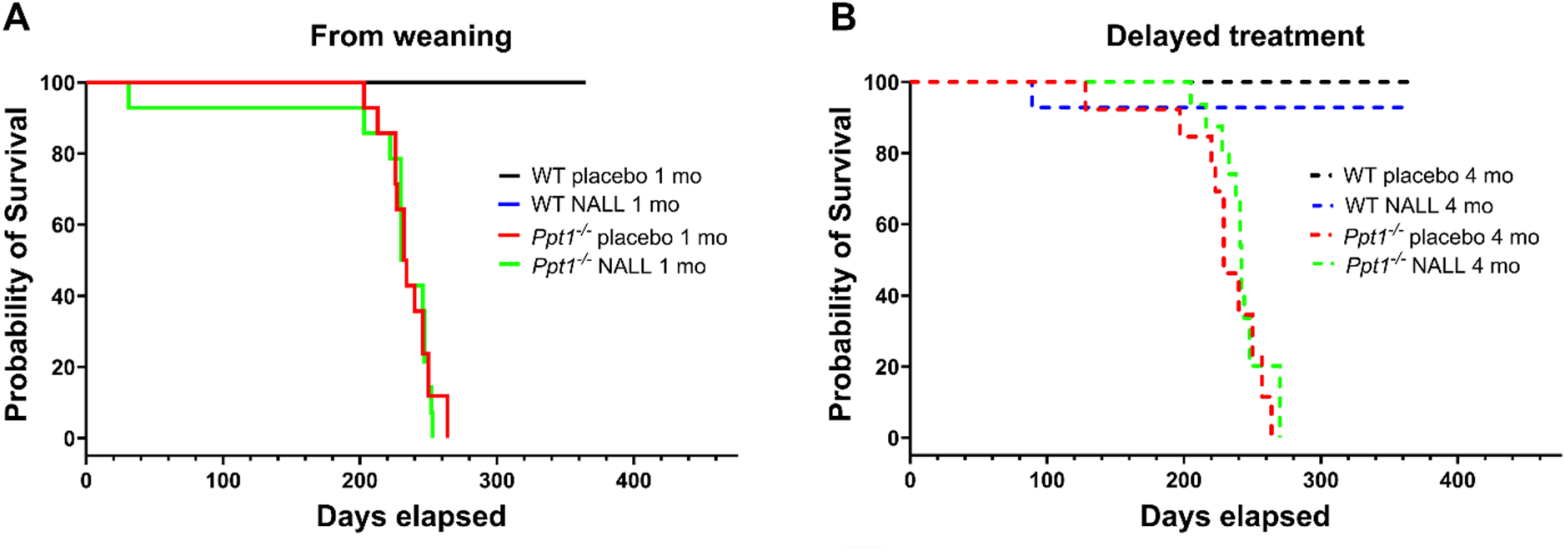
NALL treatment does not extend survival in *Ppt1*^*−/−*^ mice. Kaplan–Meier survival curves showing the effect of NALL treatment initiated either presymptomatically (1 month; **A**) or symptomatically (4 months; **B**) in PPT1-deficient (*Ppt1*^*−/−*^) vs. wild type (WT) mice. No survival benefit was observed in either treatment group. Statistical analysis: log-rank (Mantel–Cox) test.

To evaluate whether *N*-acetyl-L-leucine (NALL) influences disease progression in *Ppt1*^*−/−*^ mice, we first assessed survival following chronic oral administration of NALL initiated either pre-symptomatically (from weaning, Fig. 1A) or symptomatically (from 4 months of age, Fig. 1B). Consistent with previous reports^10,28^, *Ppt1*^*−/−*^ mice receiving placebo exhibited a median survival of approximately 8 months. NALL treatment initiated from weaning (Fig. 1A) did not extend survival compared to placebo-treated *Ppt1*^*−/−*^ mice. Similarly, delayed NALL administration starting at 4 months (Fig. 1B) failed to produce any significant survival benefit. WT mice in both treatment groups survived well beyond the study period. These results suggest that NALL does not modify lifespan in this CLN1 mouse model, regardless of the timing of treatment initiation.

### Motor coordination deficits persist despite NALL treatment

**Fig. 2:**
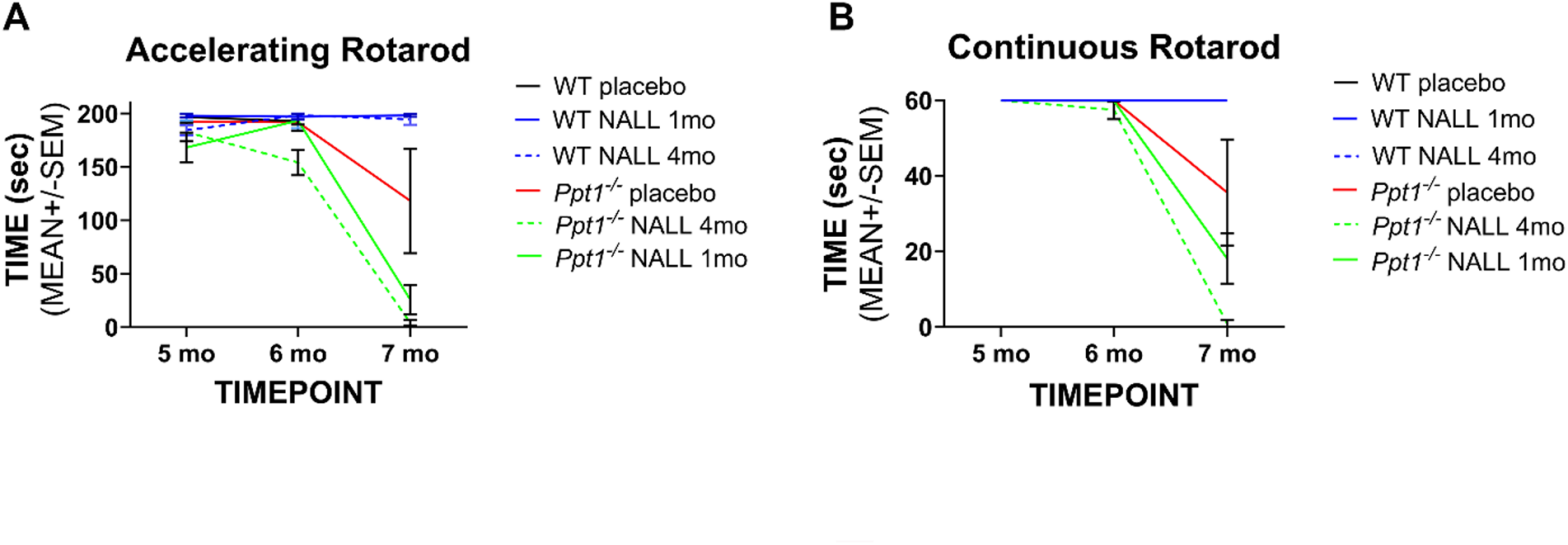
NALL treatment fails to improve motor coordination in *Ppt1*^*−/−*^ mice. (**A**) Accelerating rotarod performance measured in PPT1-deficient (*Ppt1*^*−/−*^) and wild type (WT) mice at 5, 6, and 7 months of age. (**B**) Continuous rotarod performance across the same time points. *Ppt1*^*−/−*^ mice treated with NALL from either 1 or 4 months of age did not show improvement relative to placebo-treated *Ppt1*^*−/−*^ mice. WT controls maintained consistent performance levels throughout. Data represent mean ± SEM.

Given that motor dysfunction represents a core clinical feature of CLN1 disease, and that NALL has shown particular efficacy in improving cerebellar function in other LSDs^20,23–24^, we evaluated motor performance using accelerating rotarod and continuous rotarod. On the accelerating rotarod (Fig. 2A), *Ppt1*^*−/−*^ mice demonstrated the anticipated progressive decline in performance from 5 to 7 months of age^10^, taking significantly less time to fall than WT controls. NALL-treated *Ppt1*^*−/−*^ mice showed no meaningful improvement in this task, with both early and delayed treatment groups performing similarly or worse than placebo-treated *Ppt1*^*−/−*^ mice at all time points tested. The continuous rotarod (Fig. 2B) revealed a similar pattern, with placebo-treated *Ppt1*^*−/−*^ mice showing dramatically reduced latency to fall by 7 months compared to WT controls^10^, and NALL treatment providing no protective effect or impairing rotarod performance.

### Gait abnormalities in *Ppt1*^*−/−*^ mice are widespread and progressive

**Fig. 3:**
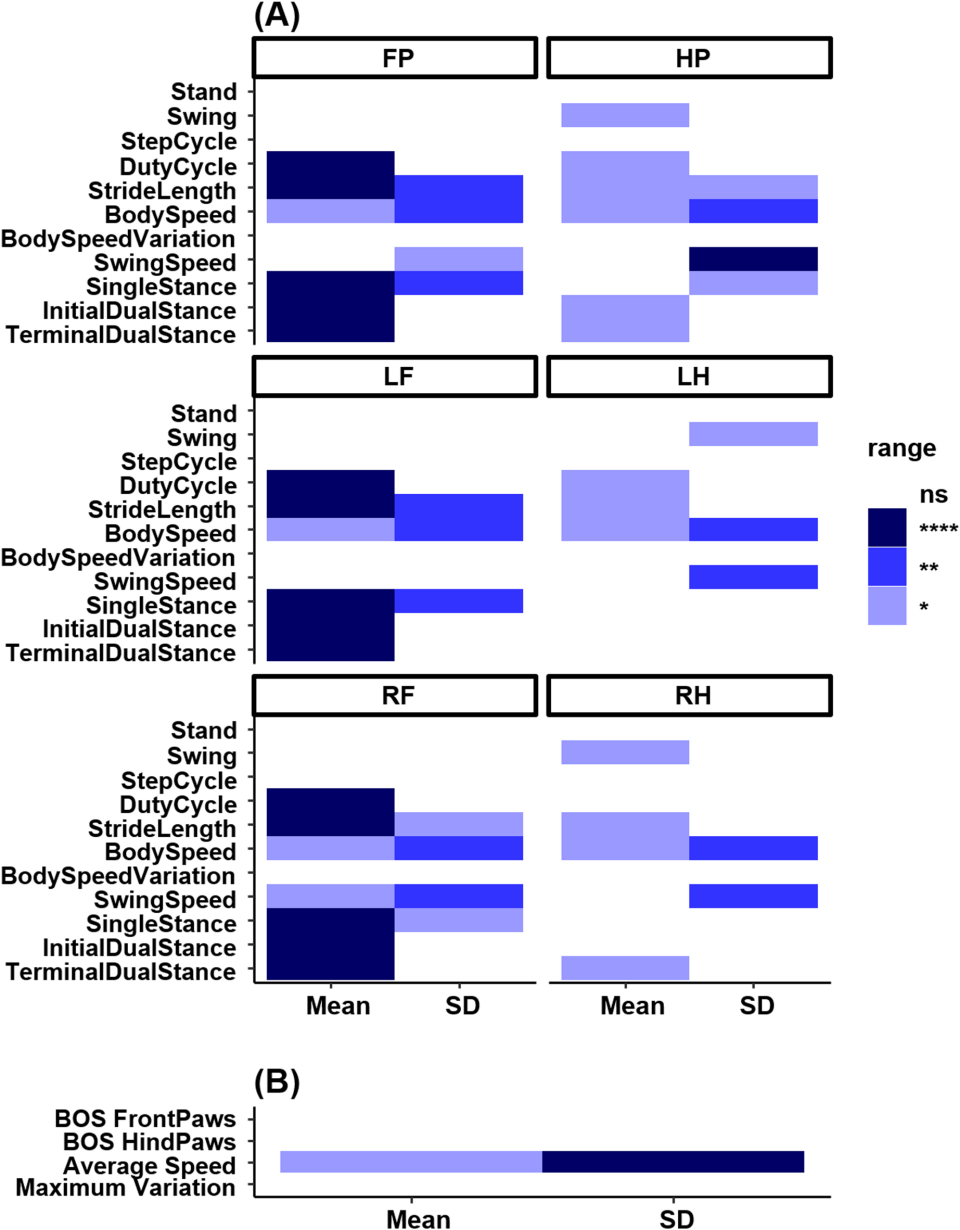
Widespread physical gait abnormalities in *Ppt1*^*−/−*^ mice. (**A**) Heatmaps displaying variables that are significantly altered in PPT1-deficient (*Ppt1*^*−/−*^) vs. wild type (WT) mice. These include significantly altered paw-wise gait variables for front paws (FP), hind paws (HP), left front (LF), left hind (LH), right front (RF), and right hind (RH). Mean and standard deviation (SD) values are shown separately. (**B**) Summary of selected non-paw-wise variables including base of support (BOS), average speed, and maximum variation. Heatmaps were generated in R version 4.5.0 (R Core Team, 2025; https://www.r-project.org) using the tidyverse package (version 2.0.0) and patchwork package (version 1.3.2). Colors indicate the level of statistical significance: *p ≤ 0.05, **p ≤ 0.01, ***p ≤ 0.001, ****p ≤ 0.0001; ns = not significant.

Automated gait analysis using the CatWalk XT system revealed extensive and progressive motor abnormalities in placebo-treated *Ppt1*^*−/−*^ mice, consistent with our previous observations^10^. Out of 138 physical gait variables assessed, 54 were significantly disrupted compared to wild type controls. These impairments included parameters related to stance, stride length, duty cycle, and variability in body speed (Fig. 3A). Although both forelimbs and hindlimbs were affected, our analyses revealed forelimb parameters were more broadly impaired, as reflected by significance across multiple metrics. Notably, the observed progression pattern in mutant mice was opposite to the typical developmental trajectory seen in WT mice. This divergence from healthy maturation provides a robust phenotypic marker for evaluating therapeutic efficacy in subsequent analyses (Fig. 3B).

### Severe inter-limb coordination deficits in *Ppt1*^*−/−*^ mice remain unaffected by NALL treatment

**Fig. 4:**
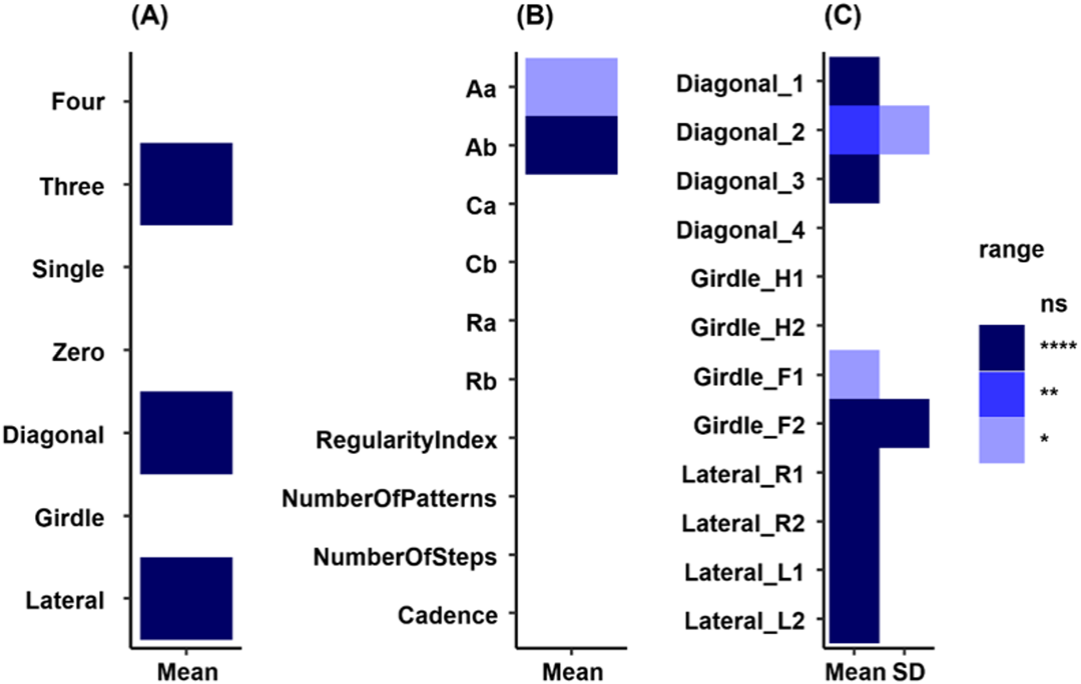
Coordination-related gait parameters are profoundly disrupted in *Ppt1*^*−/−*^ mice. (**A**–**C**) Heatmaps indicate statistically significant differences in coordination-related gait parameters between *Ppt1*^*−/−*^ and WT mice at 7 months of age. (**A**) Support metrics: percentage of time paws spent in specific paw combinations contacting the ground (e.g., diagonal, lateral, girdle). (**B**) Step sequence parameters: frequency of six predefined inter-limb footfall patterns (Aa, Ab, Ca, Cb, Ra, Rb), including the regularity index, number of steps/patterns, and cadence. (**C**) Phase dispersion/coupling parameters: temporal coordination between diagonal, girdle, and lateral paw pairs. Heatmaps were generated in R version 4.5.0 (R Core Team, 2025; https://www.r-project.org) using the tidyverse package (version 2.0.0) and patchwork package (version 1.3.2). Color intensity reflects statistical significance: *p*  ≤ 0.05, *p*  ≤ 0.01, *p*  ≤ 0.001, *p* ≤ 0.0001 (****); ns = not significant. Despite widespread abnormalities, NALL treatment (initiated at either 1 or 4 months) had no measurable effect on these coordination-related variables (data not shown).

Comprehensive analyses of 107 coordination-related gait parameters revealed significant disruption in 33 variables in placebo-treated *Ppt1*^*−/−*^ mice at 7 months of age, indicating profound motor coordination impairment. These included support metrics (e.g., diagonal and lateral paw combinations; Fig. 4A), step sequence features (e.g., predefined footfall patterns and regularity index; Fig. 4B), and phase coupling parameters (Fig. 4C). Both diagonal and lateral inter-limb coordination were markedly affected, highlighting a breakdown in both spatial and temporal gait synchrony. However, NALL treatment, whether initiated early or late, had no detectable effect on these coordination-related metrics in *Ppt1*^*−/−*^ mice, underscoring the limited therapeutic impact of NALL on inter-limb motor integration in CLN1 disease mice.

### Early NALL treatment selectively improves gait variability in *Ppt1*^*−/−*^ mice

**Fig. 5:**
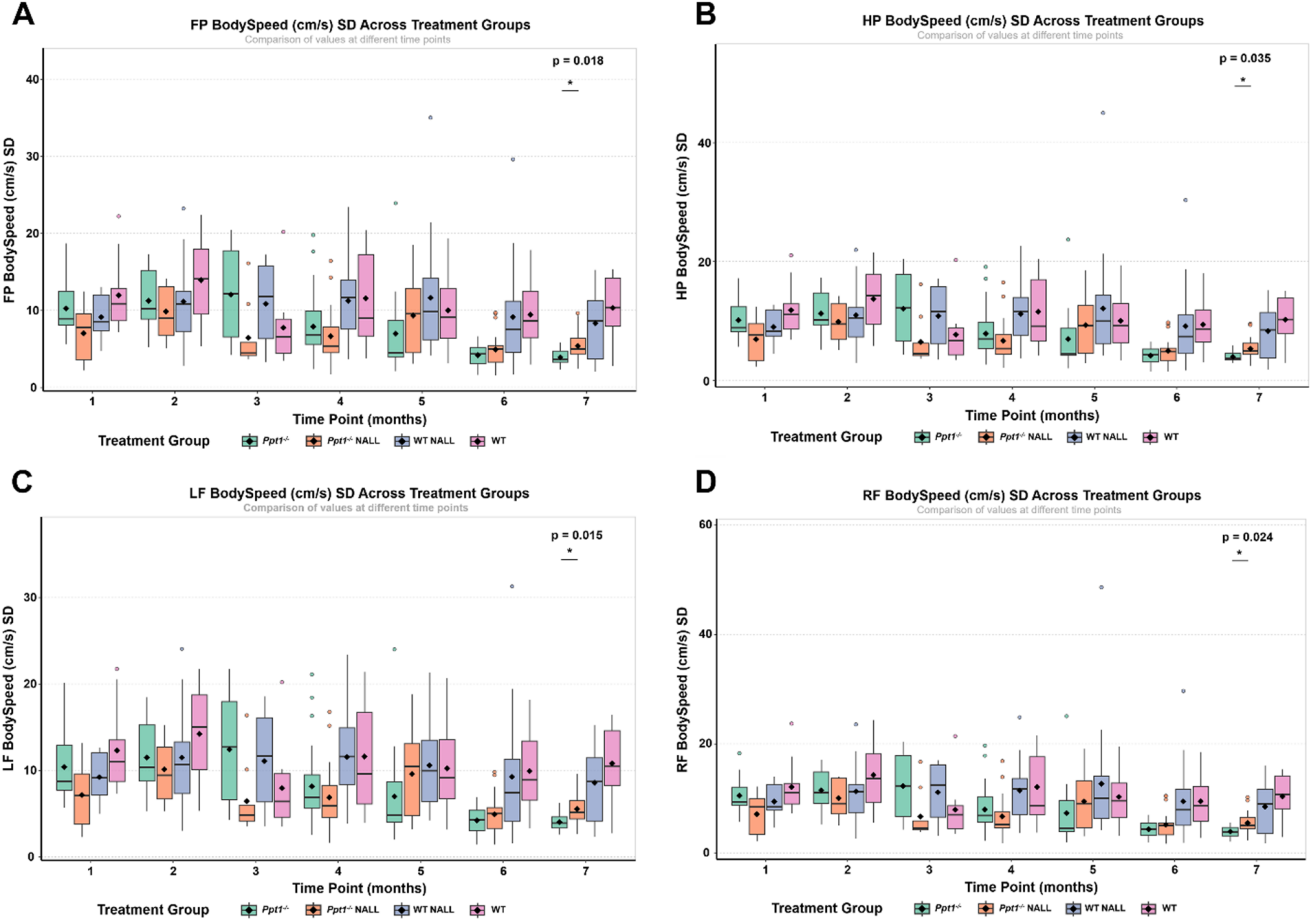
Early NALL treatment reduces locomotor variability in *Ppt1*^*−/−*^ mice. (**A**–**D**) Standard deviation (SD) of body speed for each individual paw across treatment groups at different time points, as assessed by automated gait analysis comparing PPT1-deficient (*Ppt1*^*−/−*^) vs. wild type (WT) mice. Early NALL treatment (initiated at 1 month of age) significantly reduced body speed variability in *Ppt1*^*−/−*^ mice compared to placebo controls at 7 months, specifically in the: (A) front paws (FP), (**B**) hind paws (HP), (**C**) left front (LF), and (**D**) right front (RF) paws. Each dot represents an individual mouse; box plots indicate the distribution of values within each group. Diamonds denote group means. p-values indicate significance of group comparisons at 7 months (Kruskal–Wallis).

To evaluate the efficacy of NALL on gait performance at the disease endstage, we conducted a two-way ANOVA comparing all treatment groups at 7 months of age. Out of the 138 physical gait parameters assessed, only four showed statistically significant differences between *Ppt1*^*−/−*^ mice treated with NALL from 1 month of age and their placebo-treated counterparts. These modest treatment effects were not present in symptomatic *Ppt1*^*−/−*^ mice treated with NALL from 4 months of age (data not shown). All four variables that were improved in *Ppt1*^*−/−*^ mice treated with NALL from 1 month of age related to the standard deviation (SD) of body speed for front paws (Fig. 5A), hind paws (Fig. 5B) and both front paws individually (Fig. 5C–D). Body speed represents the linear distance traveled by the mouse’s body between two consecutive contacts of the same paw, divided by the time taken to complete that step cycle. The SD of this parameter captures the step-to-step variability in locomotor rhythm and precision and WT mice show natural variability in their speed as they can modulate their speed flexibly during their normal locomotion. In contrast, *Ppt1*^*−/−*^ mice have a very slow speed with minimal variability as they are neurologically compromised and incapable of modulating their speed. As such, the increase in the SD of speed values in NALL treated *Ppt1*^*−/−*^ mice reflects functional benefit in these mice in which treatment was initiated immediately postweaning, but was absent if this treatment initiation was delayed to 4 months of age when *Ppt1*^*−/−*^ mice are symptomatic.

In placebo-treated *Ppt1*^*−/−*^ mice, SD values were significantly lower compared to wild type controls, indicating impaired locomotor output. Early, but not delayed, NALL treatment, NALL treatment, significantly increased this variability in all four limbs (*p* < 0.05), suggesting a partial and age-dependent rescue of rhythmicity and step regularity with more variable speeds of locomotion. Despite these improvements, no significant treatment effects were observed in other physical gait parameters such as stride length, swing duration, stance phase, or duty cycle, which only showed non-significant trends toward normalization. Furthermore, no changes were detected in coordination-related parameters, such as phase coupling, step sequence regularity, or inter-limb timing (see Fig. 4), reinforcing that the therapeutic effect of NALL in *Ppt1*^*−/−*^ mice is restricted to improving within-limb motor consistency, without restoring higher-order coordination between limbs.

These findings demonstrate that early intervention with NALL confers a modest but quantifiable benefit in locomotor stability and variation in speed in *Ppt1*^*−/−*^ mice, but this benefit does not extend to the temporal or spatial coordination deficits that characterize the advanced gait phenotype of CLN1 disease mice.

### NALL fails to reduce neuroinflammation or storage burden in thalamocortical regions

**Fig. 6:**
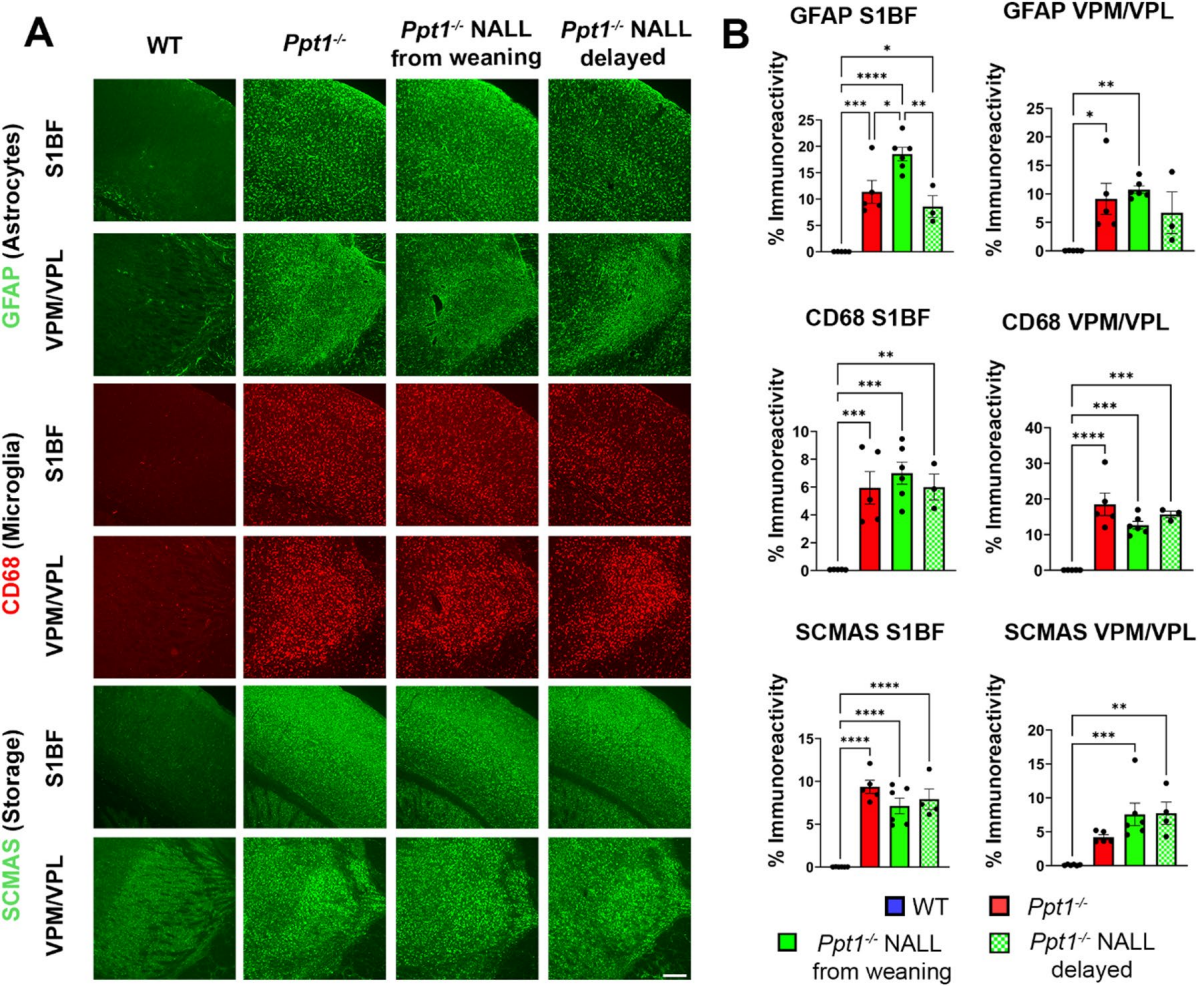
NALL does not attenuate glial activation or storage burden in thalamocortical regions of *Ppt1*^*−/−*^ mice. (**A**) Representative images of SCMAS, CD68 and GFAP staining. (**B**) Quantification of CD68, GFAP, and SCMAS immunoreactivity in the primary somatosensory cortex (S1BF) and ventral thalamic nuclei (VPM/VPL) of PPT1-deficient (*Ppt1*^*−/−*^) and wild type (WT) mice at 7 months of age. Data represent mean ± SEM; *p < 0.05, **p < 0.01, ***p < 0.001, ****p < 0.0001; ns = not significant (two-way ANOVA with Bonferroni post hoc test). Scale bar = 200 μm.

To examine the potential effects of NALL upon neuropathological changes, we performed immunohistochemical analysis for markers of microglial activation (CD68), astrocytosis (GFAP), and storage material accumulation (SCMAS) in the primary somatosensory cortex (S1BF) and thalamic relay nuclei (VPL/VPM). These are phenotypes and structures that are well-defined features of murine CLN1 disease and often used as outcome measures to judge therapeutic efficacy in NCL mouse models^10,28–31^. As shown in Fig. 6A–B, placebo-treated *Ppt1*^*−/−*^ mice exhibited pronounced astrocytosis and microglial activation and storage accumulation compared to WT controls, characterized by elevated CD68 and GFAP immunoreactivity and robust SCMAS immunoreactivity. Quantification confirmed significant increases in all three markers across both brain regions in placebo-treated *Ppt1*^*−/−*^ mice. However, NALL treatment, whether initiated at weaning or at 4 months of age, did not reduce the level of CD68⁺ or GFAP⁺ immunoreactivity present in either the cortex or thalamus of *Ppt1*^*−/−*^ mice. Similarly, SCMAS accumulation remained unaffected by NALL treatment, regardless of when it was started. These data indicate that NALL fails to attenuate the neuroimmune response or lysosomal pathology in thalamocortical pathways of *Ppt1*^*−/−*^ mice, even when administered prior to peak disease onset.

## Discussion

This study provides a systematic evaluation of NALL treatment in a well-characterized CLN1 disease mouse model^3–9^, addressing both early and late intervention strategies. To our knowledge, this is the first report investigating NALL treatment in any NCL model. Despite previously reported treatment effects of NALL in other lysosomal storage disorders, such as Niemann–Pick type C (NPC)^15,17–23^ and GM2 gangliosidosis^32–33^, NALL failed to produce significant therapeutic benefit in *Ppt1*^*−/−*^ mice, regardless of age at treatment initiation. Our findings demonstrate that chronic oral NALL treatment neither improves neuropathological hallmarks of CLN1 disease nor alters survival, while conferring a modest but quantifiable benefit in locomotor stability with early intervention. These results highlight the challenges of generalizing therapeutic efficacy across distinct lysosomal diseases and underscore the importance of disease-specific pathomechanistic considerations.

Based on pre-clinical data in other neurodegenerative conditions^24,34^, including other LSDs^15,23^, NALL has been considered an attractive candidate small molecule for treating CNS disorders. The bioavailability of orally administered NALL supports this notion, with NALL being taken up into tissues, including the CNS, at high capacity^15–16^. This uptake is via ubiquitously expressed membrane-spanning monocarboxylate transporters (MCTs)^15–16^, resulting in elevated cytoplasmic levels of NALL within the CNS, to cause improved ATP production and ameliorated lysosomal and mitochondrial function^15,21^. More recently, NALL has been shown to additionally have a lysosome-specific mode of action in vitro^15^, promoting the translocation of TFEB into the nucleus to upregulate genes involved in lysosomal function and biogenesis^15,35–37^. This mode of action depends upon the N-acetylation of L-leucine, which in its unacetylated form did not activate TFEB^15^, with N-acetyl D-leucine proving similarly ineffective and even antagonistic to the effects of NALL.

Given the well-established role of TFEB as a key regulator of genes important for lysosomal function^38^, it might be anticipated that NALL-mediated stimulation of TFEB would also have positive effects in *Ppt1*^*−/−*^ mice. However, evidence that NALL may stimulate TFEB expression is currently limited to cultured cells^15^, and is yet to be demonstrated in any form of NCL, or in vivo. Nevertheless, our data in the present study strongly suggest that NALL administration has very little impact upon key measures of murine CLN1 disease and the contrast with effects in other LSDs is particularly striking. In NPC and GM2 models^15,17–23,32–33^, and in clinical studies^24,39^ NALL has been associated with improved motor function, reduced neuroinflammation, and partial attenuation of disease progression^20–21^, effects that were substantiated in clinical testing of NALL in NPC patients^23^. In determining whether NALL would also provide any therapeutic benefit in NCL mice, we could have chosen a mouse model of any of the three major forms, in all of which we have well-defined outcome measures. For logistical reasons we opted to obtain proof of concept data in *Ppt1*^*−/−*^ mice (CLN1 disease), rather than either *Cln2*^*R207X*^ mice (CLN2 disease) that have a severe and fatal seizure phenotype^29^ or *Cln3*^*Δex7/8*^ mice (CLN3 disease) that have a normal lifespan^40^. In *Ppt1*^*−/−*^ mice, however, neither presymptomatic nor symptomatic initiation of NALL administration altered the trajectory of glial activation, lysosomal storage accumulation, or lifespan. Although a modest reduction in gait variability was observed in early treated *Ppt1*^*−/−*^ mice, specifically, a decrease in the standard deviation of body speed across all four limbs, these changes did not extend to other gait parameters of coordination, rotarod performance, or other functional metrics. Indeed, even these modest effects upon gait speed variability were absent if NALL administration was delayed to when *Ppt1*^*−/−*^ mice are symptomatic. Notably, coordination deficits, which closely reflect cerebellar and thalamocortical dysfunction in CLN1 disease^5–6,31^, remained entirely unaltered in NALL-treated *Ppt1*^*−/−*^ mice. Similarly, no effects of NALL administration were evident upon key neuropathological outcome measures in *Ppt1*^*−/−*^ mice^3,31^. Their persistence here suggests that NALL’s mechanism of action, presumed to involve modulation of glucose metabolism, oxidative stress, and neuronal excitability^16,21^, may be insufficient to impact the primary neurodegenerative cascade in CLN1 disease.

It is also worth noting that NALL treatment had no measurable effect on survival, a critical endpoint in CLN1 disease research. Despite well-tolerated oral administration and efficient blood–brain barrier penetration of NALL^15–16^, the absence of lifespan extension suggests that even subtle functional improvements were inadequate to alter the disease course. Although brain tissue concentrations were not measured in this study, altered pharmacokinetics in the CLN1 disease-affected brain, due to blood–brain barrier disruption or glial pathology^31^, could theoretically reduce drug bioavailability or target engagement. Future pharmacokinetic and pharmacodynamic studies will be necessary to fully resolve this possibility.

The disconnect between the efficacy of NALL monotherapy in other LSDs and *Ppt1*^*−/−*^ mice may lie in differences in their pathogenesis, or in whether NALL targets the specific mechanisms that operate in each disorder. Given that the mechanisms by which NALL also exerts its effects remain poorly understood, determining the reasons why some LSDs appear to respond to NALL (e.g. NPC and Sandhoff diseases), while others may be non-responders (e.g. CLN1 disease) also remains difficult. The aggressive onset and progression of CLN1 disease may further limit the therapeutic window for interventions that act indirectly or primarily on secondary cascades downstream of PPT1-deficiency.

These negative results should not be interpreted as a dismissal of NALL’s therapeutic potential in general but rather as a demonstration of its limited value as a monotherapy in CLN1 disease. This lack of NALL’s treatment effect in *Ppt1*^*−/−*^ mice was at the same dose used successfully in NPC and Sandhoff mice after extensive range-dosing studies, and that is approved for clinical use in NPC patients. Indeed, the lack of effect of this clinically relevant dose in CLN1 disease as an early onset and rapidly progressing disorder contrasts with its success in other settings^15,17–23,32−33^ and emphasizes the need for therapies that directly correct the underlying enzymatic defect in CLN1 disease. Gene therapy and CNS-directed enzyme replacement have shown considerable promise in preclinical CLN1 models^10–14^, and it is within such contexts that adjunctive compounds like NALL might be re-evaluated. Additionally, assessing its potential benefits in peripheral organs, retina, or behavioral domains not covered in the current study may provide a more complete picture of its therapeutic reach. While our study used a single, previously validated dose that is in clinical use in NPC patients^20^, additional dose-response studies that we could not justify on animal welfare grounds, or alternative delivery routes, such as intrathecal administration, may uncover effects not evident here.

In summary, our data show that NALL, despite prior success in related diseases^15,17–23,32−33^, fails to provide significant benefit in a mouse model of CLN1 disease. These findings reinforce the critical need for rigorous disease-specific validation in preclinical studies and argue against the assumption that broadly acting neuroprotective agents will show uniform efficacy across diverse LSDs with different pathogenic cascades. Progress in CLN1 disease will likely require treatments that directly restore PPT1 function or intervene directly at the earliest pathological events. Until such therapies are realized, adjunctive agents like NALL should be considered cautiously and perhaps only within well-designed combination paradigms. As the effects of NALL are multi-factorial it is not yet possible to predict which neurological diseases may benefit from its mechanism(s) of action. As such, identifying diseases that are minimally responsive to the drug may aid in determining its MOA in diseases in which it is beneficial. This study therefore also underscores why a more comprehensive understanding of the underlying pathophysiology of CLN1 disease is needed.

## Supplementary Information

Below is the link to the electronic supplementary material.

Supplementary Material 1

## Data Availability

The raw data supporting the findings and conclusions of this study are provided in a supporting data file, or are available upon request.
